# The impact of environmental conditions on non-communicable diseases in sub-Saharan Africa: A scoping review of epidemiologic evidence

**DOI:** 10.7189/jogh.14.04003

**Published:** 2024-03-01

**Authors:** Chima Anyanwu, Jean C Bikomeye, Kirsten MM Beyer

**Affiliations:** Institute for Health and Equity, Medical College of Wisconsin, Milwaukee, Wisconsin, USA

## Abstract

**Background:**

The burden of non-communicable diseases (NCDs) in sub-Saharan Africa (SSA) is increasing. Environmental conditions such as heavy metals and air pollution have been linked with the incidence and mortality of chronic diseases such as cancer, as well as cardiovascular and respiratory diseases. We aimed to scope the current state of evidence on the impact of environmental conditions on NCDs in SSA.

**Methods:**

We conducted a scoping review to identify environmental conditions linked with NCDs in SSA by identifying studies published from January 1986 through February 2023. We searched African Index Medicus, Ovid Medline, Scopus, Web of Science, and Greenfile. Using the PICOS study selection criteria, we identified studies conducted in SSA focussed on physical environmental exposures and incidence, prevalence, and mortality of NCDs. We included only epidemiologic or quantitative studies.

**Results:**

We identified 6754 articles from electronic database searches; only 36 met our inclusion criteria and were qualitatively synthesised. Two studies were conducted in multiple SSA countries, while 34 were conducted across ten countries in SSA. Air pollution (58.3%) was the most common type of environmental exposure reported, followed by exposure to dust (19.4%), meteorological variables (13.8%), heavy metals (2.7%), soil radioactivity (2.7%), and neighbourhood greenness (2.7%). The examined NCDs included respiratory diseases (69.4%), cancer (2.7%), stroke (5.5%), diabetes (2.7%), and two or more chronic diseases (19.4%). The study results suggest an association between environmental exposures and NCDs, particularly for respiratory diseases. Only seven studies found a null association between environmental conditions and chronic diseases.

**Conclusions:**

There is a growing body of research on environmental conditions and chronic diseases in the SSA region. Although some cities in SSA have started implementing environmental monitoring and control measures, there remain high levels of environmental pollution. Investment can focus on improving environmental control measures and disease surveillance.

Non-communicable diseases (NCDs) pose a significant global burden, accounting for 41 million deaths or 74% of global mortality each year [1]. Sub-Saharan Africa (SSA) is undergoing a gradual shift from infectious diseases to NCDs such as cancer, cardiovascular and respiratory diseases, and diabetes. For example, the Global Burden of Disease 2019 study reported that the proportion of disability-adjusted life-years attributable to NCDs in SSA increased from 18.6% in 1990 to 29.8% in 2017, while communicable diseases declined, particularly from 2005 onwards [2]. In fact, mortality and morbidity attributable to NCDs in SSA are projected to surpass infectious diseases in 2030 [3]. This increasing burden of NCDs in SSA has been attributed to population growth, changes in lifestyle, and environmental conditions due to urbanisation [2,4,5].

Environmental issues related to urbanisation include unplanned sanitation infrastructure, toxic waste, air pollution, flooding, and limited investments in mitigation of and/or adaptation to adverse impacts of climate change [6-8]. In high-income countries, exposure to environmental conditions such as air pollution have been linked to several NCDs, including cardiovascular diseases, respiratory diseases, and cancer [9,10]. Furthermore, reports have suggested that proximity to hazardous waste sites could exacerbate respiratory conditions such as asthma [11,12], while flooding may disrupt and adversely impact ecological determinants of health, including housing and food security [7,13,14]. In SSA, the intersecting effects of poor environmental conditions, climate change, communicable diseases, and NCDs may worsen health outcomes and negatively impact health systems [15]. For example, malaria can affect blood pressure and increase the risk of hypertension [16,17], which has also been linked with air pollution exposure [18]. Severe climate-related disasters, such as floods and drought are common in SSA [19]. Both flood and drought have been found to increase the risk of food insecurity and malnutrition, which are both linked to chronic diseases [20].

In SSA, efforts and investments to reduce NCDs are relatively low and inadequate to address the related risk [15]. Simultaneously, there is a high prevalence of environmental risk factors that can cause NCDs in SSA [6,7,21]. Unfortunately, there is limited data and studies on environmental conditions and NCDs in SSA. Previous work, including reviews, has been limited in scope, with most studies focussing on a single environmental condition such as air pollution or flood exposure. In this scoping review, we sought to gather and synthesise epidemiologic evidence of any environmental exposure with an impact on incidence, prevalence, and/or mortality of NCDs in SSA.

## METHODS

We conducted this review in accordance with the Joanna Briggs Institute (JBI) methodology for scoping reviews [22]. We also developed a protocol for this review.

### Search Strategy

We conducted literature searches in February 2023 using African Index Medicus, Ovid Medline, Scopus, Web of Science, and Greenfile to identify relevant articles published from January 1986 to the search date. With the assistance of a medical librarian, we developed a search strategy comprising keywords related to non-communicable diseases, environment, climate, and SSA, such as ‘climate,’ ‘chemical exposure,’ ‘environment,’ ‘weather,’ ‘flood,’ ‘cancer,’ ‘stroke,’ ‘cardiovascular,’ ‘diabetes,’ ‘respiratory,’ ‘incidence,’ ‘epidemiology,’ ‘Africa South of the Sahara’, and ‘Sub-Saharan Africa’ (**Online Supplementary Document**). We only considered studies published in the English language.

We exported the search results into the Rayyan online tool [23] for literature reviews and systematic reviews to enhance the article selection process and ease collaboration between the reviewers.

### Data extraction and article selection

We used the PICOS [24] model to design the search strategy and criteria for inclusion and exclusion of studies in the scoping review:

- P (Population): Any SSA population or country;
- I (Intervention/Exposure): Any direct physical environmental exposure including flooding, air pollution, water pollution, etc. Excluding proxy measures of exposure such as proximity/frequency in the use of biomass cookstoves;
- C (Comparison): Any type of comparison, including none;
- O (Outcome): Incidence, prevalence, and/or mortality of non-infectious diseases/NCDs (including cancer, all forms of cardiovascular diseases, all forms of respiratory diseases, diabetes, all forms of chronic kidney diseases, all forms of mental illnesses, injuries, etc.). This excluded communicable diseases like HIV/AIDS, sexually transmitted infections, etc.;
- S (Study design): Empirical studies only (including case control studies, cohort studies, randomised controlled trials (RCTs), and cross-sectional studies). We excluded reviews, protocols, letters to the editor, or perspectives/opinion articles.

After deduplication, two reviewers (CA and JCB) independently screened article titles and abstracts based on these criteria. Next, the full texts of included articles were independently assessed by both reviewers for relevance. All conflicts were resolved through discussion.

### Eligibility criteria

We included articles that had keywords related to any type of physical environmental exposure; any type of epidemiologic and empirical study; any type of NCDs; and any SSA country or population in their title, abstract, or full text.

We excluded studies with a sole focus on any communicable or infectious disease and any non-SSA country or population; studies using proxy measures of environmental exposure such as proximity to point sources of pollution and frequency of the usage of biomass cooking fuel; and review articles and fully qualitative investigations.

## RESULTS

**Figure 1:**
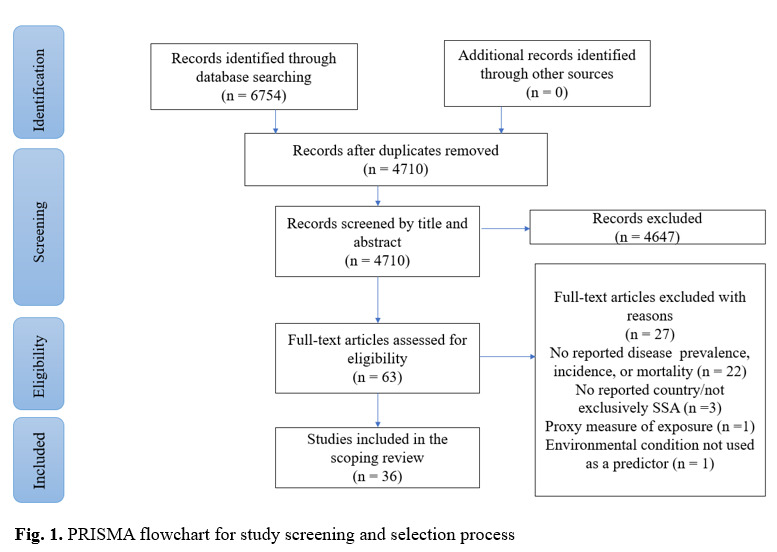
PRISMA flowchart for study screening and selection process.

We identified 6754 articles through electronic database searches (**Figure 1**) and removed 2044 duplicates. We excluded 4647 articles through title and abstract screening, due to study design (e.g. reviews, proxy measure of exposure). After the full-text screening, 27 articles were further excluded due to no reported country, absence of environmental condition, usage of proxy measure of exposure, and absence of the outcome of interest which was any NCD incidence, or prevalence and/or mortality. Only 36 articles met the inclusion criteria and were qualitatively synthesised. Data were charted following the extant literature [4], and reviewed for accuracy by one author (KB). Items charted from each study include, study characteristics, NCD, environmental condition, and disease outcome.

### Study characteristics

Most of the studies were published between 2017 and 2021 (41.6%). Almost all studies were conducted across ten SSA countries, while two were conducted in more than two SSA country. South Africa had the highest number of studies (30.5%), while Zambia had the lowest (2.7%). The studies adopted a case-control (2.7%), case-crossover (11.1%), cohort (5.5%), cross-sectional (72.2%), RCT (2.7%), and time-series (5.5%) design (**Table 1**).

**Table 1:** Summary of the characteristics of included studies

	n (%)
**Year of publication**	
1986 to 2006	4 (11.1)
2007 to 2011	5 (13.8)
2012 to 2016	6 (16.6)
2017 to 2021	15 (41.6)
2022 to February 2023	6 (16.6)
**Countries represented**	
Cameroon	2 (5.5)
Congo	2 (5.5)
Ethiopia	3 (8.3)
Malawi	2 (5.5)
Mozambique	2 (5.5)
Nigeria	5 (13.8)
Senegal	2 (5.5)
South Africa	11 (30.5)
Tanzania	4 (11.1)
Zambia	1 (2.7)
Multi-country study	2 (5.5)
**Study design**	
Case-control	1 (2.7)
Case-crossover	4 (11.1)
Cohort	2 (5.5)
Cross-sectional	26 (72.2)
Randomised controlled trial	1 (2.7)
Time series	2 (5.5)

### NCD, environmental condition, and diseases outcome

In view of NCDs, most studies (69.4%) reported on respiratory diseases, including asthma, lung cancer, influenza, acute respiratory infections, and respiratory symptoms. The remaining studies reported on at least two or more chronic diseases such as hypertension, cardiovascular and respiratory diseases, diabetes, and total cholesterol (19.4%); cancer (other than lung cancer) (2.7%); stroke (5.5%); and diabetes (2.7%). Regarding environment conditions or exposures, the included studies reported on air pollution (58.3%), dust (19.4%), temperature, relative humidity, and precipitation (13.8%), heavy metals (2.7%), neighbourhood greenness (2.7%), and soil radioactivity (2.7%). The outcomes reported in the studies included disease prevalence (50.0%), incidence (30.5%), and mortality (16.6%). One study included both incidence and prevalence as outcome (2.7%) (**Table 2**).

**Table 2:** Summary of the type of NCD, environmental conditions, and reported outcomes in terms of prevalence, incidence, and mortality

	n (%)
**Non-communicable disease**	
Respiratory diseases*	25 (69.4)
Cancer (other than lung cancer)	1 (2.7)
Stroke	2 (5.5)
Diabetes	1 (2.7)
Two or more chronic diseases†	7 (19.4)
**Environmental conditions**	
Air pollution	21 (58.3)
Dust (including paper and silica dust)	7 (19.4)
Heavy metals	1 (2.7)
Soil radioactivity	1 (2.7)
Neighbourhood greenness	1 (2.7)
Meteorological variables‡	5 (13.8)
**Disease outcomes**	
Mortality	6 (16.6)
Incidence	11 (30.5)
Prevalence	18 (50.0)
Incidence and prevalence	1 (2.7)

*Including lung cancer, asthma, influenza, acute respiratory infections, and respiratory symptoms.

†Including cardiovascular diseases, respiratory diseases, cerebrovascular diseases, diabetes, hypertension, and total cholesterol.

‡Including temperature, rainfall, relative humidity, and precipitation.

## DISCUSSION

In this scoping review, we identified 36 studies conducted in SSA and focussed on the impact of environmental conditions on NCDs.

Eleven studies were conducted in South Africa between 1986 and 2022 [25-35]. Six used a cross-sectional design [25,26,28,29,31,35], two were cohort studies [33,34], and three used case-crossover study design [27,30,32]. Eight studies reported on the impact of air pollution exposure [25-32], two reported on silica dust [34,35], while only one focussed on the impact of air temperature [33]. However, all eleven were focussed on respiratory diseases; seven reported only one type of respiratory disease [26,28,29,32–35], while four reported on two or more types of NCDs [25,27,30,31]. Out of the eleven studies conducted in South Africa, seven reported on disease mortality [25,27,30,31,33,34], three on prevalence [26,28,35], and only one on incidence [29]. The findings from the studies conducted in South Africa suggests air pollution and silica dust significantly impacts NCDs [25–32,35]. Eight studies reported a significant association between air pollution and chronic diseases [25–32], while one [36] found no association between air pollution (PM_2.5_, PM_10_, NO_2_, SO_2_, O_3_) and self-reported respiratory symptoms. This null association may be due to the small sample size (n = 233) and the assumption that air pollution estimates around monitoring stations represent city-wide exposures. One study [33] found a negative association between monthly average air temperature (Summer and Winter) and age-standardised mortality rates for chronic respiratory diseases. Another study found no significant association between silica and lung cancer, but detected a positive association between silicosis of the hilar gland and lung cancer [34].

Among other studies in SSA, five were conducted in Nigeria between 2006 and 2022; all adopted a cross-sectional design. Only one study reported on cancer (other than lung cancer), while four reported on respiratory diseases. Among these five studies, one reported on soil radioactivity as environmental condition, while four reported on air pollution as environmental exposure, including two studies reporting on indoor air pollution exposure. Three studies reported on prevalence and two on incidences. Study results from Nigeria found a significant association between air pollution and respiratory disease [37–39], but one found no significant relationship between indoor air pollution (SO_2_, NO_2_) and respiratory disease, except for CO and asthma prevalence [40]. Another study found that radiation exposure due to soil radioactivity is linked with reported cancer incidence, constituting between 1.3% and 9.2% of the total reported cases [41].

Four studies were conducted in Tanzania between 2007 and to 2018. One was a time-series analysis, while three had a cross-sectional design. All four studies were focussed on the prevalence of respiratory diseases, including chronic obstructive pulmonary disease, bronchitis, acute respiratory infections, and respiratory symptoms such as cough. Three studies focussed on the impact of dust exposure and the prevalence of respiratory diseases, while one study reported on the impact of indoor and outdoor air pollution (PM_10_, NO_2_, CO) on respiratory diseases. One study from Tanzania suggests higher levels of indoor air pollution and an association between respiratory diseases and pollution from domestic biomass fuel smoke [42]. Meanwhile, studies focussed on the impact of dust exposure found higher prevalence of respiratory diseases among the study population. For example, one found a higher prevalence of cough and chest tightness among workers in a coffee processing plant who were exposed to dust [43]. In a time-series analysis that compared the respiratory health of cement workers before and after the establishment of dust control measures, Tungu et al. (2014) [44] observed lower dust exposures among cement workers, lower prevalence of respiratory symptoms, chronic obstructive pulmonary disease, and improved lung function after introduction of dust control measures. Another study found a higher prevalence of respiratory symptoms among miners in a goldmine [45].

Three studies (one case-control and two cross-sectional) were conducted in Ethiopia between 2011 and 2023. All three focussed on the prevalence of respiratory diseases. Two examined dust exposure, while only one study investigated air pollution (PM_2.5_, PM_10_, CO2, NO2, SO2) exposure. The results from the studies conducted in Ethiopia suggests higher level of dust exposure exceeding the local threshold limit of 10 mg/m^3^ for total dust and higher levels of air pollution (150 mg/m^3^ for particulate matter) [46,47]. Results from all studies conducted in Ethiopia found dust exposure and air pollution are linked with respiratory diseases [46–48]. Moreover, one cross-sectional study conducted in Zambia suggest a link between ambient air pollution (PM_2.5_, PM_10_) and respiratory symptoms [49].

Two studies were conducted in Cameroon between 2016 and 2017. One found a median precipitation level of 154mm and a temperature of 77°F (25°C) were linked with the prevalence of diabetes [50]. Another study conducted in Cameroon using a time-series study design observed no association among temperature, rainfall, humidity, and influenza [51]. However, two studies conducted in Congo found air pollution (PM_2.5_, NO_2_, SO_2_) is linked with prevalence and incidence of respiratory symptoms [52,53]. Another study conducted in Senegal also found a link between NO_2_ exposure and incidence of acute respiratory infections after adjusting for relevant cofounders [54]. Meanwhile, one cross-sectional study in Senegal also found heavy metal exposure is linked with respiratory diseases [55].

Two studies were conducted in Malawi. In one of them, Nightingale et al. (2019) [56] found 48-hour of CO exposure was linked with prevalence of respiratory symptoms. However, a RCT in rural Malawi found no significant association between daily average CO exposure among children who experienced pneumonia and those who did not [57]. This null association may be due to the overall low level of CO in their assessment. In addition, this study did not assess PM, a common pollutant linked with respiratory diseases.

Although poor environmental conditions and exposures are linked with chronic diseases [9,21], certain health benefits may be derived from the natural environment such as exposure to greenery [58,59]. For example, a multi-country study conducted in Tanzania, Uganda, and South Africa among 1178 participants using the Normalized Difference Vegetation Index (NDVI) found that exposure to neighborhood greenness was associated with lower body mass index (BMI), lower odds of obesity (odds ratio (OR) = 0.73; 95% confidence interval (CI) = 0.62, 0.85) and lower odds of diabetes (OR = 0.77; 95% CI = 0.62, 0.96) [60].

Two studies conducted in Mozambique found temperature declines (2.4°C to 3.0°C or 36.32°F to 37.4°F) were associated with incidence of stroke hospitalisations [61,62]. In another study conducted in 21 countries of SSA, Cai et al. (2021) [63] found prior month average air pollution (PM_2.5_) exposure was not associated with cough and acute lower respiratory infections among children under five years of age. This null association may be due to several reasons. First, a global PM_2.5_ model may not have captured local emissions that are linked with biomass fuel burning in rural areas. Second, exposures were assigned at survey clusters of participants locations (coordinates were displaced by 2–10 km of participants residence). Third, self-reporting of respiratory disease outcomes may have led to recall bias.

In this review, 30 studies found an association between environmental conditions and various types of chronic diseases, suggesting a link between environmental conditions and NCDs, particularly respiratory diseases in SSA. Air pollution, particularly with PM_2.5_, was the most assessed environmental exposure among the included studies. Most of the studies examining the impact of air pollution on chronic diseases reported high levels of air pollution (PM_2.5_) exceeding the World Health Organization’s previous and current air quality guidelines of 10 μg/m^3^ and 5 μg/m^3^ for PM_2.5_ [64]. The highest range for PM_2.5_ (120-973 μg/m^3^) was reported in one study conducted in Ethiopia [46]. PM_2.5_ is a common pollutant and is linked with respiratory symptoms, cancer, and cardiovascular diseases [9,63,65,66]. Air pollution is reported as a major cause of NCD mortality in SSA and was responsible for 1.1 million deaths across the region in 2019 [21].

Dust and meteorological variables were the other leading environmental conditions reported in our scoping review. The included studies suggest temperature declines are associated with stroke and occupational dust exposure is linked with respiratory symptoms in SSA. This suggests a need for increased environmental monitoring and health tracking programs in SSA. Although certain SSA countries such as South Africa and Tanzania have started implementing environmental control measures, there still exist higher levels of environmental pollution in many of SSA countries. Future investments might focus on cleaner energies, particularly in rural parts of SSA. There is also need for improved diseases surveillance and more comprehensive health-related databases in SSA.

### Limitations

This review has some limitations. First, many of the included studies used self-reported diseases outcome, which may have increased the chances of recall bias, i.e. participants may not have remembered important details regarding symptoms of past diseases. Second, exposure misclassification may have occurred; many of the studies assessing certain environmental exposures such as air pollution assume the variables measured at a few sites represented exposure to participants and for the larger geographic location. Third, we restricted this review to only studies published in English.

## CONCLUSIONS

There is a modest, but growing body of research on environmental conditions and chronic diseases in SSA. This scoping review presented the existing evidence of the impact of environmental exposures on NCDs in SSA. Air pollution and respiratory diseases were the major environmental conditions and NCDs reported in studies assessed in this review. Our findings suggest an association between environmental conditions and NCDs, particularly respiratory diseases; we hope this study will provide guidance to researchers, policy makers, and international agencies to build upon existing research that will improve environmental health and reduce the burden of NCDs in SSA.

## Additional material


Online Supplementary Document
