# Supplementary material for: The impact of environmental conditions on non-communicable diseases in sub-Saharan Africa: A scoping review of epidemiologic evidence
Source: J Glob Health. 2024 Mar 1;14:04003. doi: 10.7189/jogh.14.04003 (PMC10902803; doi:10.7189/jogh.14.04003)
Supplement: Online Supplementary Document [file jogh-14-04003-s001.pdf]

Supplementary Document

Preferred Reporting Items for Systematic reviews and Meta-Analyses extension for Scoping Reviews (PRISMA-ScR) Checklist

SECTION	ITEM	PRISMA-ScR CHECKLIST ITEM	REPORTED ON PAGE #
**TITLE**			
Title	1	Identify the report as a scoping review.	1
**ABSTRACT**			
Structured summary	2	Provide a structured summary that includes (as applicable): background, objectives, eligibility criteria, sources of evidence, charting methods, results, and conclusions that relate to the review questions and objectives.	1-3
**INTRODUCTION**			
Rationale	3	Describe the rationale for the review in the context of what is already known. Explain why the review questions/objectives lend themselves to a scoping review approach.	2
Objectives	4	Provide an explicit statement of the questions and objectives being addressed with reference to their key elements (e.g., population or participants, concepts, and context) or other relevant key elements used to conceptualize the review questions and/or objectives.	3
**METHODS**			
Protocol and registration	5	Indicate whether a review protocol exists; state if and where it can be accessed (e.g., a Web address); and if available, provide registration information, including the registration number.	Supplementary document
Eligibility criteria	6	Specify characteristics of the sources of evidence used as eligibility criteria (e.g., years considered, language, and publication status), and provide a rationale.	3
Information sources*	7	Describe all information sources in the search (e.g., databases with dates of coverage and contact with authors to identify additional sources), as well as the date the most recent search was executed.	2
Search	8	Present the full electronic search strategy for at least 1 database, including any limits used, such that it could be repeated.	Supplementary document
Selection of sources of evidence†	9	State the process for selecting sources of evidence (i.e., screening and eligibility) included in the scoping review.	3-4
Data charting process‡	10	Describe the methods of charting data from the included sources of evidence (e.g., calibrated forms or forms that have been tested by the team before their use, and whether data charting was done independently or in duplicate) and any processes	3-4

SECTION	ITEM	PRISMA-ScR CHECKLIST ITEM	REPORTED ON PAGE #
		for obtaining and confirming data from investigators.	
Data items	11	List and define all variables for which data were sought and any assumptions and simplifications made.	4
Critical appraisal of individual sources of evidence§	12	If done, provide a rationale for conducting a critical appraisal of included sources of evidence; describe the methods used and how this information was used in any data synthesis (if appropriate).	n/a
Synthesis of results	13	Describe the methods of handling and summarizing the data that were charted.	3-4
**RESULTS**			
Selection of sources of evidence	14	Give numbers of sources of evidence screened, assessed for eligibility, and included in the review, with reasons for exclusions at each stage, ideally using a flow diagram.	2-3; Fig.1
Characteristics of sources of evidence	15	For each source of evidence, present characteristics for which data were charted and provide the citations.	Tables 1-2
Critical appraisal within sources of evidence	16	If done, present data on critical appraisal of included sources of evidence (see item 12).	n/a
Results of individual sources of evidence	17	For each included source of evidence, present the relevant data that were charted that relate to the review questions and objectives.	Tables 1-5
Synthesis of results	18	Summarize and/or present the charting results as they relate to the review questions and objectives.	4-6
**DISCUSSION**			
Summary of evidence	19	Summarize the main results (including an overview of concepts, themes, and types of evidence available), link to the review questions and objectives, and consider the relevance to key groups.	4-6
Limitations	20	Discuss the limitations of the scoping review process.	6
Conclusions	21	Provide a general interpretation of the results with respect to the review questions and objectives, as well as potential implications and/or next steps.	7
**FUNDING**			
Funding	22	Describe sources of funding for the included sources of evidence, as well as sources of funding for the scoping review. Describe the role of the funders of the scoping review.	7

JBI = Joanna Briggs Institute; PRISMA-ScR = Preferred Reporting Items for Systematic reviews and Meta-Analyses extension for Scoping Reviews.

* Where *sources of evidence* (see second footnote) are compiled from, such as bibliographic databases, social media platforms, and Web sites.

† A more inclusive/heterogeneous term used to account for the different types of evidence or data sources (e.g., quantitative and/or qualitative research, expert opinion, and policy documents) that may be eligible in a scoping review as opposed to only studies. This is not to be confused with *information sources* (see first footnote).
‡ The frameworks by Arksey and O'Malley (6) and Levac and colleagues (7) and the JBI guidance (4, 5) refer to the process of data extraction in a scoping review as data charting.
§ The process of systematically examining research evidence to assess its validity, results, and relevance before using it to inform a decision. This term is used for items 12 and 19 instead of "risk of bias" (which is more applicable to systematic reviews of interventions) to include and acknowledge the various sources of evidence that may be used in a scoping review (e.g., quantitative and/or qualitative research, expert opinion, and policy document).

From: Tricco AC, Lillie E, Zarin W, O'Brien KK, Colquhoun H, Levac D, et al. PRISMA Extension for Scoping Reviews (PRISMAScR): Checklist and Explanation. Ann Intern Med. 2018;169:467–473. doi: 10.7326/M18-0850.

Study Protocol

Topic: The Impact of Environmental Conditions on Non-Communicable Diseases in Sub-Saharan Africa: A Scoping Review of Epidemiologic Evidence

Authors
Chima Anyanwu [1], Jean C. Bikomeye [1], Kirsten Beyer [1]

[1] Institute for Health and Equity, Medical College of Wisconsin, Milwaukee, Wisconsin, USA

Abstract

Objective: To review and synthesize epidemiologic evidence of any environmental exposure with an impact on incidence, prevalence, and/or mortality of non-communicable diseases (NCD) in Sub-Saharan Africa (SSA).

Introduction: The burden of non-communicable diseases (NCD) is increasing in Sub-Saharan Africa (SSA). Environmental conditions such as heavy metals and air pollution have been linked with incidence and mortality of chronic diseases such as cancer, cardiovascular and respiratory diseases (RD). Environmental exposures can increase the risk of chronic diseases.

Inclusion criteria: The Population Intervention/Exposure Comparison Outcome Study Design (PICOS) will be used for data extraction. **The inclusion criteria include,** any SSA population or country (P), any direct physical environmental exposure (I/E), any type of comparison (C), any reported incidence, prevalence, and mortality of NCDs (O), and only empirical studies (S)

Methods: Database searches will be conducted using African Index Medicus, Ovid Medline, Scopus, Web of Science, and Greenfile without start date restriction through February 2023. The search will be restricted to only studies published in English. Only epidemiologic or quantitative studies will be included.

Dissemination: The results will be disseminated through a peer-reviewed publication

Introduction

The burden of non-communicable diseases (NCD) is increasing in Sub-Saharan Africa (SSA). Environmental conditions such as heavy metals and air pollution have been linked with incidence and mortality of chronic diseases such as cancer, cardiovascular and respiratory diseases (RD) in high income countries [1,2]. Mortality and morbidity attributable to chronic diseases in SSA are projected to surpass infectious diseases in 2030 [3]. The projection is due to poor environmental conditions such as high levels of air pollution, flooding, unplanned sanitation infrastructure and limited investments in mitigation of and/or adaptation to adverse impacts of climate change [4–6]. In SSA, efforts and investments to reduce NCDs are relatively low and inadequate to address risks [7]. Previous work, including reviews, has been limited in scope and most studies have focused on a single environmental condition such as air pollution or flood exposure. The objective of this scoping review is to review and synthesize epidemiologic evidence of any environmental exposure with an impact on incidence, prevalence, and/or mortality of NCDs in SSA.

Eligibility Criteria

Inclusion criteria for relevant articles are: the presence of certain keywords related to any type of physical environmental exposure, any type of epidemiologic and empirical study, any type of NCDs, and any SSA country or population. The specified keywords will be identified in the title, abstract, or full text.

Exclusion criteria will include a sole focus on any communicable or infectious disease, and any non-SSA country or population. Studies using proxy measures of environmental exposure such as proximity to point sources of pollution, and frequency of the usage of biomass cooking fuel will be excluded. Review articles and fully qualitative investigations will also be excluded.

Types of Sources

This scoping review will consider epidemiologic and quantitative studies including experimental and quasi-experimental study designs such as randomized controlled trials, non-randomized controlled trials, before and after studies and interrupted time-series studies. In addition, analytical observational studies including prospective and retrospective cohort studies, case-control studies and analytical cross-sectional studies will be considered for inclusion. This review will also consider descriptive observational study designs including case series, individual case reports and descriptive cross-sectional studies for inclusion. No qualitative study will be included.

Methods

The proposed scoping review will be conducted in accordance with the JBI methodology for scoping reviews [8]

Search Strategy

The search strategy will aim to locate both published and unpublished studies. Literature searches will be conducted without start date restriction through February 2023 using African Index Medicus, Ovid Medline, Scopus, Web of Science, and Greenfile to identify relevant articles. With the assistance of a reference medical librarian, certain search keywords related to non-communicable diseases, environment, climate, and Sub-Saharan Africa will be used. Sample search keywords will include “climate”, “chemical exposure”, “environment”, “weather”, “flood”, “cancer”, “stroke”, “cardiovascular”, “diabetes”, “respiratory”, “incidence”, “epidemiology”, “Africa South of the Sahara”, “Sub-Saharan Africa”. Only studies published in English language will be included. The search results will be exported to Rayyan online tool to enhance the article selection process and ease collaboration between the reviewers. Duplicates will be removed. There will be two reviewers who will independently screen the article titles and abstracts based on the study inclusion and exclusion criteria, and conflicts will be resolved by discussion until consensus was reached. The full texts of included articles will be independently assessed by both reviewers for their relevance. All conflicts were resolved by discussion.

Data Extraction and Article Selection

The PICOS [9] model will be used to design the search strategy and criteria for inclusion and exclusion of studies in the scoping review.

**P (population)**	Any Sub-Saharan Africa population (or country)
**I/E (Intervention/Exposure)**	Any direct physical environmental exposure including flooding, air pollution, water pollution, etc. Excluding proxy measures of exposure such as proximity/frequency in the use of biomass cookstoves
**C (Comparison)**	Any type of comparison, including none
**O (Outcome)**	Incidence, prevalence, and/or mortality of Non-infectious/Non-communicable diseases (including cancer, all forms of cardiovascular diseases, all forms of respiratory diseases, and diabetes, all forms of chronic kidney diseases, all forms of mental illnesses, injuries, etc.). Exclude communicable diseases like HIV/AIDS, sexually transmitted infections, etc.
**S (Study design)**	Empirical studies only (including case control studies, cohort studies, randomized controlled trials, and cross-sectional studies). Reviews, protocols, letters to the editor, or perspectives/opinion articles will be excluded

Data Analysis and Presentation

Data will be charted following the extant literature [10], and according to the Preferred Reporting Items for Systematic Reviews and Meta-Analyses extension for Scoping Reviews (PRISMA-ScR). The charted data will be reviewed for accuracy by one author. Items that will be charted from each study will include, study characteristics, NCD, environmental condition, and disease outcome. The evidence from included studies will be summarized.

Funding
None

Conflicts of Interest
None

References

1 Rodopoulou S, Samoli E, Chalbot M-CG, Kavouras IG. Air pollution and cardiovascular and respiratory emergency visits in Central Arkansas: A time-series analysis. Sci Total Environ. 2015;536:872–9. Medline:26232212 doi:10.1016/j.scitotenv.2015.06.056

2 Coleman NC, Burnett RT, Higbee JD, Lefler JS, Merrill RM, Ezzati M, et al. Cancer mortality risk, fine particulate air pollution, and smoking in a large, representative cohort of US adults. Cancer Causes Control. 2020;31:767–76. Medline:32462559 doi:10.1007/s10552-020-01317-w

3 Holmes MD, Dalal S, Volmink J, Adebamowo CA, Njelekela M, Fawzi WW, et al. Non-communicable diseases in sub-Saharan Africa: the case for cohort studies. PLoS Med. 2010;7:e1000244. Medline:20485489 doi:10.1371/journal.pmed.1000244

4 Opoku SK, Filho WL, Hubert F, Adejumo O. Climate Change and Health Preparedness in Africa: Analysing Trends in Six African Countries. Int J Environ Res Public Health. 2021;18:4672. Medline:33925753 doi:10.3390/ijerph18094672

5 Suhr F, Steinert JI. Epidemiology of floods in sub-Saharan Africa: a systematic review of health outcomes. BMC Public Health. 2022;22:268. doi:10.1186/s12889-022-12584-4

6 Glenn BE, Espira LM, Larson MC, Larson PS. Ambient air pollution and non-communicable respiratory illness in sub-Saharan Africa: a systematic review of the literature. Environmental Health. 2022;21:40. doi:10.1186/s12940-022-00852-0

7 Li Z, Shi J, Li N, Wang M, Jin Y, Zheng Z. Temporal trends in the burden of non-communicable diseases in countries with the highest malaria burden, 1990–2019: Evaluating the double burden of non-communicable and communicable diseases in epidemiological transition. Globalization and Health. 2022;18:90. doi:10.1186/s12992-022-00882-w

8 Peters MDJ, Godfrey C, McInerney P, Khalil H, Larsen P, Marnie C, et al. Best practice guidance and reporting items for the development of scoping review protocols. JBI Evidence Synthesis. 2022;20:953. doi:10.11124/JBIES-21-00242

9 Nang C, Piano B, Lewis A, Lycett K, Woodhouse M. Using The PICOS Model To Design And Conduct A Systematic Search: A Speech Pathology Case Study. :51.

10 Mudie K, Jin MM, Tan, Kendall L, Addo J, dos-Santos-Silva I, et al. Non-communicable diseases in sub-Saharan Africa: a scoping review of large cohort studies. Journal of Global Health. 2019;9:020409. doi:10.7189/jogh.09.020409.

Ovid Medline search terms and results

1	("Africa South of the Sahara*" or Sub-Saharan Africa or Subsaharan Africa or Angola* or Benin or Edo or Botswana* or Burkina Faso or Burkinabe or Burundi* or Cameroon* or Central African Republic or Chad or Chadian* or Congo* or Comoros or Comoran* or Cote d'Ivoire or Ivorian* or Democratic Republic of the Congo or Equatorial Guinea or Equatoguinean* or Eritrea* or Ethiopia* or Gabon* or Gambia* or Ghana* or Guinea-Bissau or Bissau-Guinean* or Kenya* or Lesotho or Mosotho* or Basotho* or Liberia* or Madagascar or Malagas* or Malawi* or Mali or Mauritania* or Mozambi* or Namibia* or Niger* or Nigeria* or Republic of Congo or Rwanda* or "Sao Tome and Principe" or Sao Tomean* or Senegal* or Sierra Leone* or Somalia* or South Africa* or Sudan* or Swaziland or Swazi* or Tanzania* or Togo* or Uganda* or United Republic of Tanzania or Zambia* or Zimbabwe*).mp. or exp "Africa South of the Sahara"/	380435
2	(exp environment/ or exp environmental health/ or environment*.mp.) not ((built environment* or social environment* or work environment*).mp. or exp built environment/ or exp environment design/)	2573100
3	exp environmental pollution/ or exp environmental exposure/ or exp air pollution/	618335
4	exp climate/ or exp climatic processes/ or exp air movements/ or exp climate change/ or exp cyclonic storms/ or exp droughts/ or exp floods/ or exp greenhouse effect/ or exp tidal waves/ or exp weather/	724975
5	(climate* or storm* or drought* or flood* or greenhouse or tidal wave* or weather or pollut* or (environment* adj3 expos*) or (chemical* adj3 expos*)).mp.	773757
6	2 or 3 or 4 or 5	2967947
7	exp respiratory tract diseases/ or exp lung diseases/ or exp pleural diseases/ or exp thoracic diseases/ or exp asthma/ or exp Noncommunicable Diseases/	1686116
8	exp cardiovascular diseases/ or exp heart diseases/ or exp vascular diseases/ or exp stroke/ or exp diabetes mellitus/ or exp neoplasms/	6628021
9	((disease* adj3 (noncommunicable or respirat* or lung* or pleural or thoracic or cardiovascular or heart or vascular)) or (stroke* or diabet* or neoplas* or cancer* or asthma*)).mp.	5893645
10	7 or 8 or 9	8898629
11	1 and 6 and 10	4618
12	limit 11 to english language	4344
13	seroprevalence or seroincidence or seroepidemiol* or screening).mp. or exp epidemiologic methods/ or exp epidemiologic studies/ or exp sentinel surveillance/ or exp seroepidemiologic studies/ or exp cohort studies/ or exp cross-sectional studies/ or exp longitudinal studies/ or exp follow-up studies/ or exp prospective studies/	9287794
14	12 and 13	3425
15	limit 14 to (letter or "review")	319
16	14 not 15	3106
	https://access.ovid.com/custom/redirector/index.html?dest=https://go.openathens.net/redirector/mcw.edu?url=http://ovidsp.ovid.com/ovidweb.cgi?T=JS&NEWS=N&PAGE=main&SHAREDSEARCHID=WaOGkinWepomcEng5CK97U5gNO1YUcruS4ExPpc3vTNtXPFXPtZTEQyCLDorGmDg	

Scopus search terms and results

TITLE-ABS-KEY (climate* OR storm* OR drought* OR flood* OR greenhouse OR "tidal wave*" OR weather OR pollut* OR (environment* W/3 expos*) OR (chemical W/3 expos*))

TITLE-ABS-KEY (environment* AND NOT ("built environment*" OR "social environment*" OR "work environment*"))

TITLE-ABS-KEY (("Africa South of the Sahara*" OR "Sub-Saharan Africa" OR "Subsaharan Africa" OR angola* OR benin OR edo OR botswana* OR "Burkina Faso" OR burkinabe OR burundi* OR cameroon* OR "Central African Republic" OR chad OR chadian* OR congo* OR comoros OR comoran* OR "Cote d'Ivoire" OR ivorian* OR "Democratic Republic of the Congo" OR "Equatorial Guinea" OR equatoguinean* OR eritrea* OR ethiopia* OR gabon* OR gambia* OR ghana* OR "Guinea-Bissau" OR "Bissau-Guinean*" OR kenya* OR lesotho OR mosotho* OR basotho* OR liberia* OR madagascar OR malagas* OR malawi* OR mali OR mauritania* OR mozambi* OR namibia* OR niger* OR nigeria* OR "Republic of Congo" OR rwanda* OR "Sao Tome and Principe" OR "Sao Tomean*" OR senegal* OR "Sierra Leone*" OR somalia* OR "South Africa*" OR sudan* OR swaziland OR swazi* OR tanzania* OR togo* OR uganda* OR "United Republic of Tanzania" OR zambia* OR zimbabwe*))

((TITLE-ABS-KEY (climate* OR storm* OR drought* OR flood* OR greenhouse OR "tidal wave*" OR weather OR pollut* OR (environment* W/3 expos*) OR (chemical W/3 expos*))) OR (TITLE-ABS-KEY (environment* AND NOT ("built environment*" OR "social environment*" OR "work environment*")))) AND (TITLE-ABS-KEY (("Africa South of the Sahara*" OR "Sub-Saharan Africa" OR "Subsaharan Africa" OR angola* OR benin OR edo OR botswana* OR "Burkina Faso" OR burkinabe OR burundi* OR cameroon* OR "Central African Republic" OR chad OR chadian* OR congo* OR comoros OR comoran* OR "Cote d'Ivoire" OR ivorian* OR "Democratic Republic of the Congo" OR "Equatorial Guinea" OR equatoguinean* OR eritrea* OR ethiopia* OR gabon* OR gambia* OR ghana* OR "Guinea-Bissau" OR "Bissau-Guinean*" OR kenya* OR lesotho OR mosotho* OR basotho* OR liberia* OR madagascar OR malagas* OR malawi* OR mali OR mauritania* OR mozambi* OR namibia* OR niger* OR nigeria* OR "Republic of Congo" OR rwanda* OR "Sao Tome and Principe" OR "Sao Tomean*" OR senegal* OR "Sierra Leone*" OR somalia* OR "South Africa*" OR sudan* OR swaziland OR swazi* OR tanzania* OR togo* OR uganda* OR "United Republic of Tanzania" OR zambia* OR zimbabwe*)))

TITLE-ABS-KEY (((disease* W/3 (respirat* OR lung* OR pleural OR thoracic OR cardiovascular OR heart OR vascular)) OR (stroke* OR diabet* OR neoplas* OR cancer* OR asthma*)))

(TITLE-ABS-KEY (((disease* W/3 (respirat* OR lung* OR pleural OR thoracic OR cardiovascular OR heart OR vascular)) OR (stroke* OR diabet* OR neoplas* OR cancer* OR asthma*)))) AND (((TITLE-ABS-KEY (climate* OR storm* OR drought* OR flood* OR greenhouse OR "tidal wave*" OR weather OR pollut* OR (environment* W/3 expos*) OR (chemical W/3 expos*))) OR (TITLE-ABS-KEY (environment* AND NOT ("built environment*" OR "social environment*" OR "work environment*")))) AND (TITLE-ABS-KEY (("Africa South of the Sahara*" OR "Sub-Saharan Africa" OR "Subsaharan Africa" OR angola* OR benin OR edo OR botswana* OR "Burkina Faso" OR burkinabe OR burundi* OR cameroon* OR "Central African Republic" OR chad OR chadian* OR congo* OR comoros OR comoran* OR "Cote d'Ivoire" OR ivorian* OR "Democratic Republic of the Congo" OR "Equatorial Guinea" OR equatoguinean* OR eritrea* OR ethiopia* OR gabon* OR gambia* OR ghana* OR "Guinea-Bissau" OR "Bissau-Guinean*" OR kenya* OR lesotho OR mosotho* OR basotho* OR liberia* OR madagascar OR malagas* OR malawi* OR mali OR mauritania* OR mozambi* OR namibia* OR niger* OR nigeria* OR "Republic of Congo" OR rwanda* OR "Sao Tome and Principe" OR "Sao Tomean*" OR senegal* OR "Sierra Leone*" OR somalia* OR "South Africa*" OR sudan* OR swaziland OR swazi* OR tanzania* OR togo* OR uganda* OR "United Republic of Tanzania" OR zambia* OR zimbabwe*)))) AND (LIMIT-TO (LANGUAGE , "English"))

(TITLE-ABS-KEY (prevalence OR incidence OR epidemiol* OR survey OR "rapid assessment" OR "situation assessment" OR "situational assessment" OR cohort OR surveillance OR seroprevalence OR seroincidence OR seroepidemiol* OR screening OR "sentinel surveillance" OR "cross-sectional stud*" OR "longitudinal stud*" OR "follow-up stud*" OR "prospective stud*")) AND ((TITLE-ABS-KEY (((disease* W/3 (noncommunicable or respirat* OR lung* OR pleural OR thoracic OR cardiovascular OR heart OR vascular)) OR (stroke* OR diabet* OR neoplas* OR cancer* OR asthma*)))) AND (((TITLE-ABS-KEY (climate* OR storm* OR drought* OR flood* OR greenhouse OR "tidal wave*" OR weather OR pollut* OR (environment* W/3 expos*) OR (chemical W/3 expos*))) OR (TITLE-ABS-KEY (environment* AND NOT ("built environment*" OR "social environment*" OR "work environment*")))) AND (TITLE-ABS-KEY (("Africa South of the Sahara*" OR "Sub-Saharan Africa" OR "Subsaharan Africa" OR angola* OR benin OR edo OR botswana* OR "Burkina Faso" OR burkinabe OR burundi* OR cameroon* OR "Central African Republic" OR chad OR chadian* OR congo* OR comoros OR comoran* OR "Cote d'Ivoire" OR ivorian* OR "Democratic Republic of the Congo" OR "Equatorial Guinea" OR equatoguinean* OR eritrea* OR ethiopia* OR gabon* OR gambia* OR ghana* OR "Guinea-Bissau" OR "Bissau-Guinean*" OR kenya* OR lesotho OR mosotho* OR basotho* OR liberia* OR madagascar OR malagas* OR malawi* OR mali OR mauritania* OR mozambi* OR namibia* OR niger* OR nigeria* OR "Republic of Congo" OR rwanda* OR "Sao Tome and Principe" OR "Sao Tomean*" OR senegal* OR "Sierra Leone*" OR somalia* OR "South Africa*" OR sudan* OR swaziland OR swazi* OR tanzania* OR togo* OR uganda* OR "United Republic of Tanzania" OR zambia* OR zimbabwe*))))) AND (LIMIT-TO (LANGUAGE , "English")) AND (EXCLUDE (DOCTYPE , "re") OR EXCLUDE (DOCTYPE , "le"))	1866

Web of Science search terms and results

**#1 AND #7**	961	Add to query
**#2 AND #5 AND #6** and **English** (Languages) and **Review Article** or **Letter** (Exclude – Document Types)	1,893	Add to query
**TS=("Africa South of the Sahara*" OR "Sub-Saharan Africa" OR "Subsaharan Africa" OR angola* OR benin OR edo OR botswana* OR "Burkina Faso" OR burkinabe OR burundi* OR cameroon* OR "Central African Republic" OR chad OR chadian* OR congo* OR comoros OR comoran* OR "Cote d'Ivoire" OR ivorian* OR "Democratic Republic of the Congo" OR "Equatorial Guinea" OR equatoguinean***	579,728	Add to query
**#3 OR #4**	4,645,950	Add to query
**TS=(climate* OR storm* OR drought* OR flood* OR greenhouse OR "tidal wave*" OR weather OR pollut* OR (environment* NEAR/3 expos*) OR (chemical NEAR/3 expos*))**	1,788,058	Add to query
**TS=((environment*) NOT ("built environment*" OR "social environment*" OR "work environment*"))**	3,457,233	Add to query

TS=((disease* NEAR/3 (noncommunicable OR respirat* OR lung* OR pleural OR thoracic OR cardiovascular OR heart OR vascular)) OR (stroke* OR diabet* OR neoplas* OR cancer* OR asthma*))	4,622,446	Add to query
TS=((prevalence OR incidence OR epidemiol* OR survey OR "rapid assessment" OR "situation assessment" OR "situational assessment" OR cohort OR surveillance OR seroprevalence OR seroincidence OR seroepidemiol* OR screening OR "sentinel surveillance" OR "cross-sectional stud*" OR "longitudinal stud*" OR "follow-up stud*" OR "prospective stud*"))	5,204,701	Add to query

Greenfile search terms and results

#	Query	Limiters/Expanders	Last Run Via	Results
S6	S1 AND S4 AND S5	Expanders - Apply equivalent subjects<br>Search modes - Find all my search terms	Interface - EBSCOhost Research Databases<br>Search Screen - Advanced Search<br>Database - GreenFILE	376

S5	(disease* N3 (noncommunicable OR respirat* OR lung* OR pleural OR thoracic OR cardiovascular OR heart OR vascular)) OR (stroke* OR diabet* OR neoplas* OR cancer* OR asthma*)	Expanders - Apply equivalent subjects	Interface - EBSCOhost Research Databases	20,779
		Search modes - Find all my search terms	Search Screen - Advanced Search	
			Database - GreenFILE	

S4	S2 OR S3	Expanders - Apply equivalent subjects<br><br>Search modes - Find all my search terms	Interface - EBSCOhost Research Databases<br><br>Search Screen - Advanced Search<br><br>Database - GreenFILE	856,040

S3	(climate* or storm* or drought* or flood* or greenhouse or tidal wave* or weather or pollut* or (environment* N3 expos*) or (chemical* N3 expos*))	Expanders - Apply equivalent subjects	Interface - EBSCOhost Research Databases	371,063
		Search modes - Find all my search terms	Search Screen - Advanced Search	
			Database - GreenFILE	

S2	(environment* NOT ("built environment*" OR "social environment*" OR "work environment*"))	Expanders - Apply equivalent subjects	Interface - EBSCOhost Research Databases	769,754
		Search modes - Find all my search terms	Search Screen - Advanced Search	
			Database - GreenFILE	

S1	("Africa South of the Sahara*" OR "Sub-Saharan Africa" OR "Subsaharan Africa" OR angola* OR benin OR edo OR botswana* OR "Burkina Faso" OR burkinabe OR burundi* OR cameroon* OR "Central African Republic" OR chad OR chadian* OR congo* OR comoros OR comoran* OR "Cote d'Ivoire" OR ivorian* OR "Democratic Republic of the Congo" OR "Equatorial Guinea" OR equatoguinean* OR eritrea* OR ethiopia* OR gabon* OR gambia* OR ghana* OR "Guinea-Bissau" OR "Bissau-Guinean*" OR kenya* OR lesotho OR mosotho* OR basotho* OR liberia* OR madagascar OR malagas* OR malawi* OR mali OR mauritania* OR mozambi* OR namibia* OR niger* OR nigeria* OR "Republic of Congo" OR rwanda* OR "Sao Tome and Principe" OR "Sao Tomean*" OR senegal* OR "Sierra Leone*" OR somalia* OR "South Africa*" OR sudan* OR swaziland OR swazi* OR tanzania* OR togo* OR uganda* OR "United Republic of Tanzania" OR zambia* OR zimbabwe*)	Expanders - Apply<br><br>Search modes - Find all my search terms	Interface -<br><br>Search Screen - Advanced Search<br><br>Database - GreenFILE	35,634

African Index Medicus search terms and results

climate* or storm* or drought* or flood* or greenhouse or "tidal wave" or "tidal waves" or weather or pollut* or environment* or "chemical exposure" or "chemical exposures"			
noncommunicable or respirat* or lung* or pleural or thoracic or cardiovascular or heart or vascular or stroke* or diabet* or neoplas* or cancer* or asthma* or disease*			
limited to AIM and English			
tw:((tw:(climate* OR storm* OR drought* OR flood* OR greenhouse OR "tidal wave" OR "tidal waves" OR weather OR pollut* OR environment* OR "chemical exposure" OR "chemical exposures")) AND (tw:(noncommunicable OR respirat* OR lung* OR pleural OR thoracic OR cardiovascular OR heart OR vascular OR stroke* OR diabet* OR neoplas* OR cancer* OR asthma* OR disease*))) AND (collection_gim:("AIM") AND la:("en"))	445		

Characteristics of studies included.

Study	DOI	Study Design	Country	NCD	Environmental Condition	Outcome
Wyndham (1986)	https://pubmed.ncbi.nlm.nih.gov/3952586/	Cohort	South Africa	respiratory disease	Air temperature	and age specific
Hnizdo and Sluis-Cremer (1991)	DOI: 10.1136/oem.48.1.53	Cohort	South Africa	Lung cancer	Silica, tobacco smoking	Mortality
Churchyard et al. (2004)	doi: 10.1136/oem.2003.010967	Cross-sectional	South Africa	Silicosis	Silica dust	Prevalence
Farai et al. (2006)	DOI: 10.1016/j.jenvrad.2006.06.003	Cross-sectional	Nigeria	Cancer	soil radioactivity	Incidence
Kilabuko et al. (2007)		Cross-sectional	Tanzania	Acute respiratory infection	Indoor and outdoo air pollution (PM_{10}, NO_2, CO)	Prevalence
Norman et al. (2007)	DOI: 10.3390/ijerph2007010007	Cross-sectional	South Africa	Mortality and YLLs from lung cancer and cardiopulmonary disease in adults (30 years and older), and from acute respiratory infections (ARIs) in children aged 0 - 4 years.	Outdoor air pollution ($PM_{2.5,}$	Mortality
Kistnasamy et al. (2008)	doi: 10.7196/sajch.598	Cross-sectional	South Africa	Asthma among children	Air pollution (PM_{10}, Nox, SO_2)	Prevalence
Zeleke et al. (2011)	DOI: 10.1186/1471-2466-11-50	Case-control	Ethiopia	Chronic respiratory symptoms	Dust exposure	Prevalence
Sakwari et al. (2011)	DOI: 10.1186/1471-2466-11-54	Cross-sectional	Tanzania	wheezing, chest-tightness,	Dust exposure	Prevalence
Wichmann and Voyi (2012)	DOI: 10.3390/ijerph9113978	Case-crossover	South Africa	Respiratory disease, CVD, cerebrovascular disease	Outdoor air pollution (24-h average PM_{10}, SO_2, NO_2)	Mortality
Naidoo et al. (2013)	doi: 10.7196/sajch.598	Cross-sectional	South Africa	Asthma among school children	Outdoor air pollution ($PM_{2.5}$, PM_{10}, SO_2, NO_2, NO)	Prevalence
Tungu et al. (2014)	DOI: 10.1097/JOM.0000000000000057	Time-series	Tanzania	Chronic respiratory symptoms, lung function, COPD	Dust expsoure	Prevalence
Gomez et al. (2014)	DOI: 10.1016/j.jstrokecerebrovasdis.2013.02.012	Cross-sectional	Mozambique	Stroke hospitalization	relative daily humidity,	Incidence
Gomez et al. (2015)	DOI: 10.1016/j.clineuro.2014.12.002	Case crossover	Mozambique	Stroke hospitalization	relative daily humidity,	Incidence
Lontchi-Yimagou. (2016)	DOI: 10.1186/s12889-016-3090-1	Cross-sectional	Cameroon	Diabetes	precipitation and	prevalence
Monamele et al. (2017)	DOI: 10.1371/journal.pone.0186914	Time-series	Cameroon	Influenza	humidity	Incidence
Nkhama et al. (2017)	DOI: 10.3390/ijerph14111351	Cross-sectional	Zambia	Respiratory symptoms	($PM_{2.5}$, and PM_{10})	Incidence
Mbelambela et al. (2017)	DOI: 10.1186/s12199-017-0608-9	Cross-sectional	Congo	Impaired pulmonary function	SO_2)	Prevalence
Rusibamayila et al. (2018)	doi: 10.29024/aogh.2323	Cross-sectional	Tanzania	(impairment)	Dust	Prevalence
Aliyu and Botai (2018)	doi: 10.2991/j.jegh.2018.04.002	Cross-sectional	Nigeria	Respiratory symptoms	($PM_{2.5}$, and PM_{10}, CO, SO_2)	Incidence
Nightingale at al. (2019)	DOI: 10.1164/rccm.201805-0936OC	Cross-sectional	Malawi	Chronic respiratory symptoms	Air pollution ($PM_{2.5}$, CO)	Prevalence

Michellier et al. (2020)	https://doi.org/10.1186/s12940-020-00615-9	Cross-sectional	DRC	Acute respiratory symptoms	Air pollution (SO_2)	Incidence
Olaniyan et al. (2020)	DOI: 10.1016/j.envres.2020.109606	Cross-sectional	South Africa	Respiratory symptoms	Air pollution (NO_2, $PM_{2.5}$)	Incidence
Mortimer et al. (2020)	doi: 10.1016/j.chest.2020.03.064	Randomized controlled trial	Malawi	Pneumonia	Air pollution (CO)	Incidence
Oloyede et al. (2020)	https://pubmed.ncbi.nlm.nih.gov/32150634/	Cross-sectional	Nigeria	Respiratory symptoms	Air pollution ($PM_{2.5}$, PM_{10})	Prevalence
Foko et al. (2021)	DOI: 10.1016/j.admp.2021.04.008	Cross-sectional	Senegal	Respiratory symptoms	Heavy metals	Incidence
Cai et al. (2021)	DOI: 10.3390/ijerph18189729	Cross-sectional	21 SSA countries	Acute lower respiratory infection	($PM_{2.5}$)	Prevalence
Iyer et al. (2021)	DOI: 10.1016/j.envres.2020.110397	Cross-sectional	Uganda, South Africa, Tanzania	Diabetes, hypertension, obesity, total cholesterol	Neighborhood greeness	Prevalence
Thabete et al. (2021)	DOI: 10.1007/s11356-021-13778-w	Case-crossover	South Africa	Respiratory and cardiovascular diseases	Ambient air pollution(PM_{10}, NO_2, SO_2)	Mortality
Beketie et al. (2021)	DOI: 10.1007/s11869-021-01109-4	Cross-sectional	Ethiopia	Respiratory diseases	Air pollution ($PM_{2.5}$, PM_{10}, CO_2, NO_2, SO_2)	Prevalence
Kawano et al. (2022)	https://dx.doi.org/10.1186/s12889-022-12577-3	Cross-sectional	Senegal	Acute respiratory infections	Air pollution (NO_2)	Incidence
Adebayo-Ojo et al. (2022)	DOI: 10.3390/ijerph19138078	Cross-sectional	South Africa	Cardiovascular and respiratory diseases	Air pollution (PM_{10}, NO_2, SO_2, O_3)	Daily mortality
Eghomwanre and Oguntoke (2022)	DOI: 10.1007/s10661-022-10026-7	Cross-sectional	Nigeria	Asthma	Indoor air pollution (CO, SO_2, NO_2)	Prevalence
Shirinde and Wichmann (2022)	DOI: 10.1080/09603123.2022.2076813	Case-crossover	South Africa	Respiratory diseases	Air pollution (PM_{10}, NO_2, SO_2)	Mortality
Eghomwanre et al. (2022)	DOI: 10.1007/s10661-022-10135-3	Cross-sectional	Nigeria	Asthma	Indoor and outdoor air pollution (PM_1, $PM_{2.5}$, PM_{10})	Prevalence
Negash et al. (2023)	DOI: 10.1186/s12890-023-02338-2	Cross-sectional	Ethiopia	Chronic respiratory symptoms	Paper dust	Prevalence

Table 1. Summary of the characteristics of included studies

Characteristics	Studies n (%)
**Year of publication**	
1986 - 2006	4 (11.1)
2007 - 2011	5 (13.8)
2012 - 2016	6 (16.6)
2017 - 2021	15 (41.6)
2022 - February 2023	6 (16.6)
**Countries represented**	
Cameroon	2 (5.5)
Congo	2 (5.5)
Ethiopia	3 (8.3)
Malawi	2 (5.5)
Mozambique	2 (5.5)
Nigeria	5 (13.8)
Senegal	2 (5.5)
South Africa	11 (30.5)
Tanzania	4 (11.1)
Zambia	1 (2.7)
Multi-country study	2 (5.5)
**Study design**	
Case-control	1 (2.7)
Case-crossover	4 (11.1)
Cohort	2 (5.5)
Cross-sectional	26 (72.2)
Randomized controlled trial	1 (2.7)
Time series	2 (5.5)

Table 2. Summary of the type of NCD, environmental conditions, and reported outcomes in terms of prevalence, incidence, and mortality.

**Characteristics**	**n (%)**
**Non-communicable diseases**	
Respiratory diseases (Lung cancer, asthma, influenza, acute respiratory infections, respiratory symptoms)	25 (69.4)
Cancer (other than lung cancer)	1 (2.7)
Stroke	2 (5.5)
Diabetes	1 (2.7)
Two or more chronic diseases (Cardiovascular, respiratory, cerebrovascular, diabetes, hypertension, total cholesterol)	7 (19.4)
**Environmental conditions**	
Air pollution	21 (58.3)
Dust (Including paper and silica dust)	7 (19.4)
Heavy metals	1 (2.7)
Soil radioactivity	1 (2.7)
Neighborhood greenness	1 (2.7)
Meteorological variables (Temperature, rainfall, relative humidity, precipitation)	5 (13.8)
**Disease outcomes**	
Mortality	6 (16.6)
Incidence	11 (30.5)
Prevalence	18 (50.0)
Incidence and prevalence	1 (2.7)

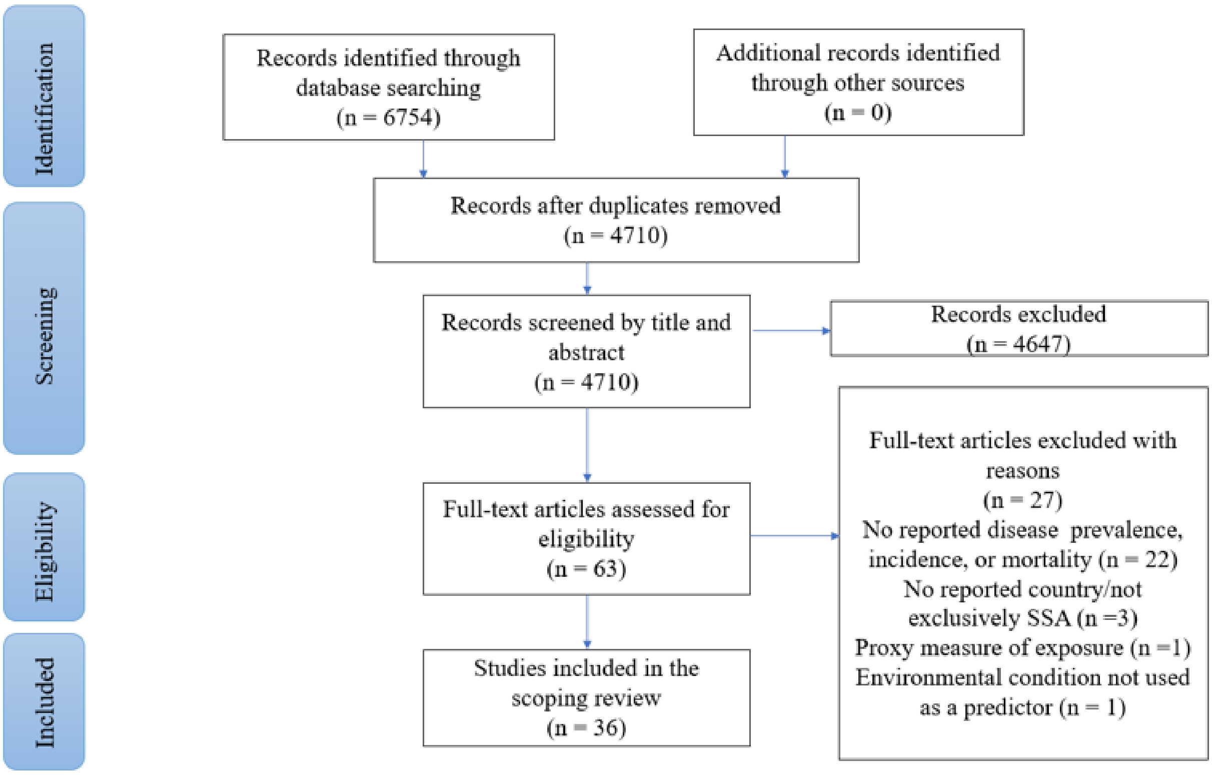

Fig. 1. PRISMA flowchart for study screening and selection process
